# The Functional Unit of *Neisseria meningitidis* 3-Deoxy-ᴅ-*Arabino*-Heptulosonate 7-Phosphate Synthase Is Dimeric

**DOI:** 10.1371/journal.pone.0145187

**Published:** 2016-02-01

**Authors:** Penelope J. Cross, Logan C. Heyes, Shiwen Zhang, Ali Reza Nazmi, Emily J. Parker

**Affiliations:** Biomolecular Interaction Centre and Department of Chemistry, University of Canterbury, Christchurch, New Zealand; University Paris Diderot-Paris 7, FRANCE

## Abstract

*Neisseria meningitidis* 3-deoxy-d-*arabino-*heptulosonate 7-phosphate synthase (*Nme*DAH7PS) adopts a homotetrameric structure consisting of an extensive and a less extensive interface. Perturbation of the less extensive interface through a single mutation of a salt bridge (Arg126-Glu27) formed at the tetramer interface of all chains resulted in a dimeric DAH7PS in solution, as determined by small angle X-ray scattering, analytical ultracentrifugation and analytical size-exclusion chromatography. The dimeric *Nme*DAH7PS^R126S^ variant was shown to be catalytically active in the aldol-like condensation reaction between d-erythrose 4-phosphate and phosphoenolpyruvate, and allosterically inhibited by l-phenylalanine to the same extent as the wild-type enzyme. The dimeric *Nme*DAH7PS^R126S^ variant exhibited a slight reduction in thermal stability by differential scanning calorimetry experiments and a slow loss of activity over time compared to the wild-type enzyme. Although *Nme*DAH7PS^R126S^ crystallised as a tetramer, like the wild-type enzyme, structural asymmetry at the less extensive interface was observed consistent with its destabilisation. The tetrameric association enabled by this Arg126-Glu27 salt-bridge appears to contribute solely to the stability of the protein, ultimately revealing that the functional unit of *Nme*DAH7PS is dimeric.

## Introduction

The shikimate pathway is responsible for the biosynthesis of important aromatic compounds including the aromatic amino acids phenylalanine (Phe), tyrosine (Tyr) and tryptophan (Trp) [1]. The presence of this pathway in plants, microorganisms and apicomplexan parasites and its absence in mammals has drawn attention to the enzymes of this pathway as antimicrobial drug design targets [2]. 3-Deoxy-d-*arabino-*heptulosonate 7-phosphate synthase (DAH7PS) catalyses the first committed step of the shikimate pathway, which involves the aldol-like condensation of d-erythrose 4-phosphate (E4P) and phosphoenolpyruvate (PEP) to form 3-deoxy-d-*arabino-*heptulosonate 7-phosphate (DAH7P). Entry into the pathway is controlled by the allosteric regulation of DAH7PS by the pathway end products Phe, Tyr and Trp, or other intermediates of the pathway [3].

Despite significant sequence diversity, all known DAH7PS enzymes share a common (β/α)_8_-barrel core and similar active site architectures formed by conserved residues that support a similar catalytic mechanism [4]. On the basis of sequence and size, DAH7PS enzymes are classified into groups (type Iα, type Iβ and type II). Each group features distinct structural elements appended to the core catalytic barrel that are associated with the allosteric inhibition of the enzymes and different quaternary structure associations [3,5–10]. The type I groups, type Iα and type Iβ, share approximately 30% identity, and although a common dimeric unit is shared between by enzymes of these groups, they form different homotetrameric structures [11].

The DAH7PS isolated from *N*. *meningitidis (Nme*DAH7PS), the causative agent of pyogenic meningitis and meningococcal septicaemia, belongs to the type Iα group. This protein adopts a homotetrameric assembly, and contains similar structural features to the other characterised type Iα DAH7PSs from *Saccharomyces cerevisiae* and *Escherichia coli*. *S*. *cerevisiae* and *E*. *coli* both express multiple isozymes of DAH7PS and the catalytic activity of each of these isozymes is sensitive to the presence of a different aromatic amino acid, however, the genome of *N*. *meningitidis*, encodes a single DAH7PS (*Nme*DAH7PS) which is most sensitive towards feedback inhibition by Phe [12].

**Fig 1:**
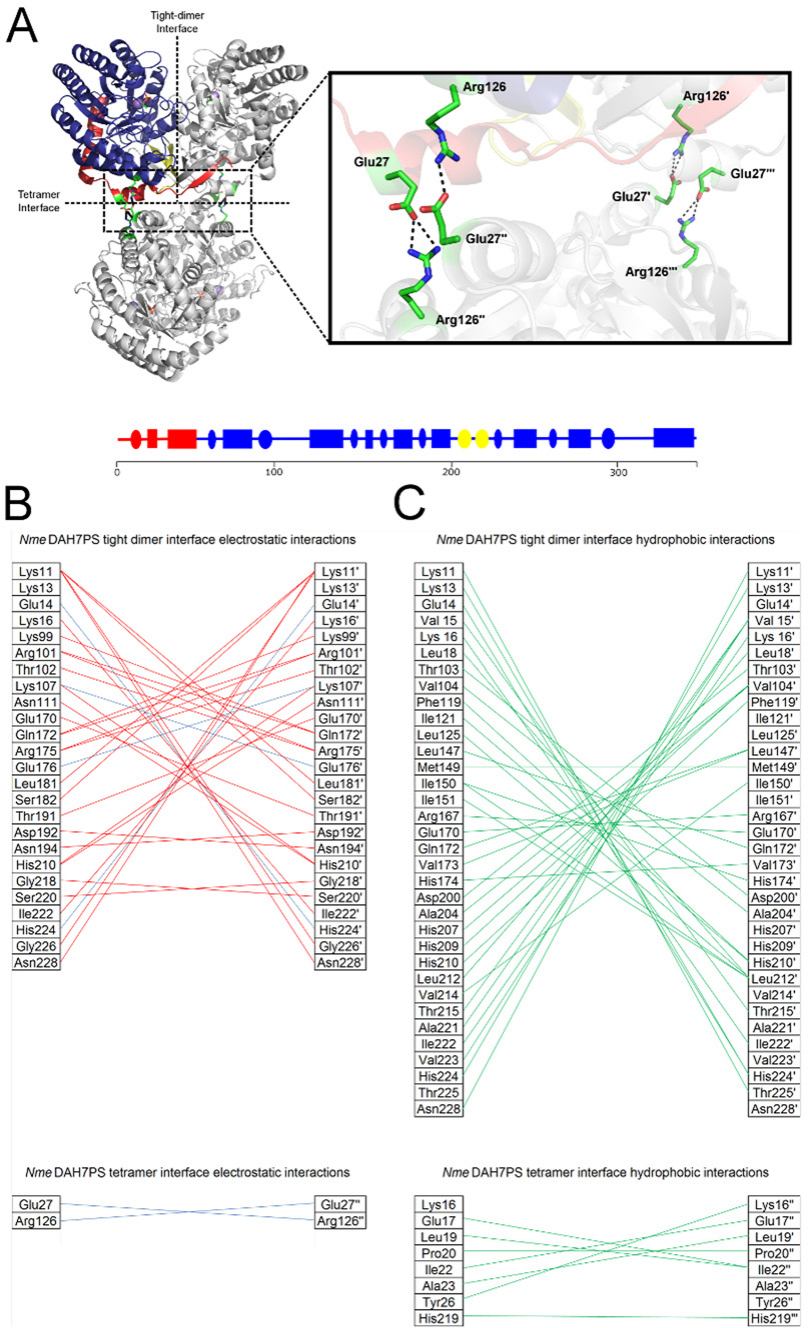
A). Quaternary structure of *Nme*DAH7PS (PDB code: 4HSN) indicating the tight dimer interface and tetramer interface. The core catalytic (β/α)_8_ barrel is coloured blue and the N-terminal and internal extensions are coloured red and yellow, respectively. Manganese metal ion (purple), ligand (PEP) and Glu27-Arg126 salt bridge (green-sticks) A schematic of secondary structural features of monomeric *Nme*DAH7PS according to residue number, circles denote β-sheets and rectangles denote α-helices B). Hydrogen bonding (red) and salt bridge (blue) interactions identified by analysis of each interface. Top. Tight-dimer. Bottom. Tetramer C) Hydrophobic interactions (green) at each interface of *Nme*DAH7PS. Top. Tight-dimer. Bottom. Tetramer. Chain B, C and D are denoted `, “and “`respectively.

A comparison of the structure of the *S*. *cerevisiae* and *E*. *coli* enzymes with *Nme*DAH7PS reveals that although the single chain and dimeric units are superimposable, the two dimeric units that generate the homotetramer are oriented at different angles. The homotetrameric *Nme*DAH7PS comprises two interfaces, the more extensive (tight dimer) and the less extensive (tetramer interface) (Fig 1). The tight dimer interface involves interactions between 79 residues per chain and buries ~ 2800 Å^2^ or 18% of the surface area between chains. The tetramer interface is far weaker and consists of 17 residues per chain and buries only ~510 Å^2^ or 3.8% of the *Nme*DAH7PS chain. Both of these interfaces are comparable to the other structurally characterised type Iα DAH7PS enzymes [13,14]. In the *Eco*DAH7PS structure (PDB code: 1QR7) the angle between tight dimers is ~22° less than that of *Nme*DAH7PS (PDB code: 4HSN). Conversely, the arrangement of tight dimers in *Sce*DAH7PS (PDB code: 1OAB) is ~26° greater than that of *Nme*DAH7PS. This illustrates the variability at the tetramer interface between members of the same DAH7PS subfamily.

Herein, we describe the disruption of the quaternary structure of *Nme*DAH7PS via mutagenesis of a single salt bridge at the tetramer interface, generating a dimeric species that we show to be the functional unit of *Nme*DAH7PS.

## Experimental Procedures

### Bacterial Strains, Plasmids, Media and Growth Conditions

#### Wild-Type *Nme*DAH7PS

Purification procedures and kinetic measurements were performed as previously described [12].

#### *Nme*DAH7PS^R126S^

The variant R126S (*Nme*DAH7PS^R126S^) was constructed using a Quik Change Lightning Mutagenesis Kit using the pT7-7 wild-type plasmid as template. Primers used to introduce the mutation were FOR: 5’-CATCAATTTCCGTTTGAGCCAGGAGAGGCAGCCTG-3’ and REV: 5’- CAGGCTGCCTCTCCTGGCTCAAACGGAAATTGATG -3’. The PCR product was transformed into Top10 cells. Plasmids of the expected size, as determined by agarose gel, were DNA sequenced to identify those containing the successful mutation and transformed into BL21 Star^TM^ (DE3) cells. Expression and purification were carried out as previously described for the wild-type enzyme [12].

### Interface Analysis

Resmap [15] and PISA [16] were used to analyse and visualise the interfacing residues. Free energy calculations for the formation of interfaces were calculated from PISA.

### Differential Scanning Calorimetry

DSC was performed on a NanoDSC (TA instruments). Protein solutions were dialysed in SEC buffer (10 mM BTP, pH 7.3, 100mM KCl and 200μM PEP) and the dialysis buffer was used as reference. A final protein concentration of 26 μM (1 mg/mL) was used for the DSC experiments, and prior to scanning, all solutions were degassed by stirring under vacuum. An excess pressure of 3 atm was applied to the cells during scanning. Buffer scans were run prior to protein scans. DSC scans were performed using a heating rate of 1°C/min, and the data was analysed using the Nanoanalyze™ software supplied with the instrument.

### Analytical Size Exclusion Chromatography

To determine the molecular mass of *Nme*DAH7P synthase in solution, protein standards of known molecular mass were applied to a Superdex S200 column (GE Healthcare). β-Amylase (200 kDa), Alcohol dehydrogenase (150 kDa), ovalbumin (75 kDa), conalbumin (66 kDa) and carbonic anhydrase (29 kDa) were used as molecular weight standards.

### Analytical Ultracentrifugation

Sedimentation velocity experiments were performed in a Beckman Coulter XL-1 analytical ultracentrifuge equipped with UV/Vis scanning optics at 20°C. Reference buffer solution (10 mM BTP, pH 7.3, 100 mM KCl, 200 μM PEP) and sample solutions were loaded into 12-mm double-sector cells with quartz windows, and mounted in an An-50 Ti 8-hole rotor.

Protein samples at concentrations of 0.2, 0.4 and 1.2 mg/mL were centrifuged at 50,000 *g* and the absorbance data was collected at 277 nm or 276 nm for *Nme*DAH7PS and *Nme*DAH7PS^R126S^ respectively. Radial scans collected between 5.8 cm and 7.2 cm with 0.003 cm increments were then analysed with the continuous size distribution model using SEDFIT [17]. The partial specific volume (***v***) of the protein samples (0.737734 mL/g), buffer density (1.005 g/mL) and buffer viscosity (0.01002 cp) were calculated using the program SEDNTERP [18].

### Small Angle X-Ray Scattering

#### SAXS measurements

Measurements were performed at the Australian Synchrotron SAXS/WAXS beamline equipped with a Pilatus detector (1M, 170 mm x 170 mm, effective pixel size, 172 x 172 μm). The wavelength of the x-rays was 1.0332 Å. The sample–detector distance was 1600 mm, which provided an *q* range of 0.01–0.565 Å^−1^ [where *q* is the magnitude of the scattering vector, which is related to the scattering angle (2θ) and the wavelength (λ) as follows: *q* = (4π/λ) sinθ].

Data were collected from a 2 mm glass capillary (Hampton Research) and the temperature was 25°C. Scattering data was collected from wild-type *Nme*DAH7PS and *Nme*DAH7PS^R126S^ (initial concentration of 7 and 12 mg/mL, respectively, prior to SEC) in the buffer (10 mM BTP, pH 7.5, 100 mM KCl, 200 μM PEP). Scattering from glucose isomerase at an initial concentration of 10 mg/ml was collected to calibrate the mass of scattering particles. Data were collected in 2 second intervals. 2D intensity plots from the peak of the SEC run were radially averaged, normalised to sample transmission and background subtracted.

#### SAXS data analysis

Scattered intensity (*I*) was plotted *versus q*. Extrapolation of the DAH7PS *I*(*q*) profiles to zero angle [*I*(0)] and comparison with that of glucose isomerase standards indicated a molecular mass consistent with the *Nme*DAH7PS tetramer or dimer. All samples were devoid of an increase in intensity at low *q* (increase is indicative of aggregation). Radius of gyration (*R*_g_) did not vary significantly over the concentrations measured, and all Guinier plots were linear for *q*·*R*_g_ < 1.3. The data sets for structural analyses were recorded with 302 data points over the range 0.0110 ≤ *q* ≤ 0.4 Å^−1^. 1D profiles were background subtracted and Guinier fits were made using PRIMUS. Indirect Fourier transform was performed using GNOM [19] to yield the function *P*(*r*), which gives both the relative probabilities of distances between scattering centers and the maximum dimension of the scattering particle *D*_max_. Theoretical scattering curves were generated from atomic coordinates and compared with experimental scattering curves using CRYSOL [20].

### Kinetics

The assay system for *Nme*DAH7PS was performed as previously described by monitoring the disappearance of PEP at 232 nm [12]. Standard assay buffer was 50 mM BTP (pH 6.8) containing MnSO_4_ (100 μM) and variable concentrations of substrates PEP and E4P. Assays were carried out at 25°C

#### *Nme*DAH7PS^R126S^

The reaction mixtures for the determination of the *K*_m_ of E4P consisted of PEP (126 μM), MnSO_4_ (100 μM) and E4P (9–140 μM), in 50 mM BTP, pH 6.8 buffer. The reaction mixtures for the determination of the *K*_m_ of PEP consisted of E4P (232 μM) and PEP (12–315 μM) in assay buffer. The reaction was initiated by the addition of *Nme*DAH7PS^R126S^ (2 μL, 1.3 mg/mL).

#### *Nme*DAH7PS^WT^

The reaction mixtures for the determination of the *K*_m_ of E4P consisted of PEP (100 μM) and E4P (10–320 μM), in assay buffer. The reaction mixtures for the determination of the *K*_m_ of PEP consisted of E4P (300 μM) and PEP (5–160 μM) in assay buffer. The reaction was initiated by the addition of *Nme*DAH7PS^WT^ (2 μL, 0.6 mg/mL). Apparent *K*_m_ and *k*_cat_ values were determined by fitting the data to the Michaelis-Menten equation using Grafit (Erithicus).

### Time Dependent Loss of Activity

Standard assays were performed which consisted of E4P (230 μM) and PEP (100 μM) in assay buffer. Assays were initiated by the addition of 2μl of ~1 mg/mL *Nme*DAH7PS^WT^ or *Nme*DAH7PS^R126S^. Assays were performed in triplicate and averaged at timepoints (0 hr, 2 hr, 4 hr, 6 hr, 18 hr and 24 hr). Both *Nme*DAH7PS^WT^ and *Nme*DAH7PS^R126S^ were diluted to 1 mg/mL at time 0 hr and kept at ~4°C for the experiment duration. There was no loss of protein after 24 hr at 1 mg/mL calculated via absorbance at 280 nm using the calculated molar extinction coefficient of 31650 M^-1^ cm^-1^ post experiment [21].

### Inhibition

Standard assays were performed which consisted of E4P (74 μM), PEP (162 μM) and the amino acid; Phe, Tyr or Trp (25 μM– 1 mM) in assay buffer. Assays were were initiated by the addition of *Nme*DAH7PS^R126S^. Assays were performed in triplicate.

### Isothermal Titration Calorimetry

Binding of *Nme*DAH7PS^WT^ and *Nme*DAH7PS^R126S^ to Phe at pH 7 was measured by ITC using a VP- ITC unit operating at 298 K (Microcal, GE Health- care). Before use, the protein was buffer exchanged against binding buffer [0.5 mM MnSO_4_ in 50 mM BTP buffer (pH 7)] and all solutions were degassed in a vacuum. Protein concentration was measured by UV absorption immediately before titrations were started. The titrations were comprised of 28 injections, one 2 μL injection followed by 27 x 10 μL injections of Phe. The initial datum point was routinely deleted to allow for diffusion of ligand across the needle tip during the equilibration period. A heat of dilution experiment was measured independently and subtracted from the integrated data before curve fitting in Origin 7.0. For the binding of *Nme*DAH7PS to Phe, 15 μM of *Nme*DAH7PS^WT^ and *Nme*DAH7PS^R126S^ were used and the syringe contained 1.2 mM Phe; and the data were fitted with the two-site sequential-binding model supplied by Micro-Cal.

### Crystallisation of *Nme*DAH7PS^R126S^

*Nme*DAH7PS^R126S^ was expressed and purified as previously described for the wild-type enzyme [12]. The resulting protein solution was concentrated to approximately 10 mg/ mL. 1 μL of enzyme solution (9–11 mg/mL) was mixed with 1 μL of crystallisation buffer containing 0.1 M Tris HCl (pH 7.3), 0.2 M trimethyl-amino-*N-*oxide (TMAO), 600 μM MnSO_4_ and 15–20% (w/v) PEG 2000MME. Crystals were grown by hanging drop vapour diffusion over 500 μL of crystallisation buffer and the crystallisation trays were incubated at 20°C. Crystals began to form in 48 hours and were fully formed within seven days. Crystals were flash frozen using liquid nitrogen in a cryoprotectant solution containing reservoir solution and 20% (v/v) PEG400.

### Crystallography and Structure Determination

An X-ray diffraction dataset was collected at the Australian Synchrotron using the MX1 beamline [22]. The datasets were integrated and processed using XDS and Aimless [23,24]. Appropriate cut-off resolution was determined via CC^1/2^≥0.5 ensuring the data was complete in the highest resolution shell [25]. Space group and unit cell parameters for *Nme*DAH7PS^R126S^ were the same as those previously identified for the wild-type enzyme (PDB code 4HSN [12]) indicating that initial phases could be obtained via molecular replacement using the original structure as a search model in Phaser MR [26]. All ligands and waters were removed from the search model (PDB code 4HSN) before molecular replacement was carried out. Refmac5 was used to generate the electron density map and this was manually analysed and refined in COOT (Table 1) [27–29]. The quality of the model was optimised by consecutive model building in COOT and refinement with Refmac5. Water molecules were added manually via interpretation of the │2*Fo-Fc│*map ensuring that they had the ability to hydrogen bond to at least one acceptor or donor. In all structures, no electron density for the 14 amino acids at the N-terminus of each *Nme*DAH7PS^R126S^ chain could be found, which is attributed to these residues being part of a highly flexible allosteric region of the protein. Molprobity was used to assess structure quality during refinement cycles and before deposition [30].

**Table 1:** Data collection and refinement statistics of *Nme*DAH7PS^R126S^ variant (PDB code: 4UCG).

	*Nme*DAH7PS^R126S^
**Data Collection**	
Crystal system; space group	Orthorhombic, *P*2_1_2_1_2_1_
**Unit cell parameters**	
*a*, *b*, *c* (Å)	78.95, 132.7, 147.7
α, β, γ (°)	90, 90, 90
Resolution range (Å)	48.03–2.00 (2.03–2.00)
Measurements	1558648
Unique reflections	105375
Redundancy	14.8
Completeness (%)	100.0 (99.9)
*I/*σ (*I*)	15.2 (1.5)
R_merge_	0.152
CC_1/2_	0.564
Wilson *B*-value (Å)^2^	28.94
Matthews coefficient	2.54
**Refinement**	
*R*_factor_	0.1801
*R*_free_	0.2200
Chain length	351
Observed number of residues	334 (Chain B & D), 335 (Chain C), 336 (Chain A)
Water molecules	821
Other (PEG’s, Mn, SO4^-^)	10
Ligand	4
**Mean *B* (Å)**^**2**^	
Protein	35.02
Water	40.5
Other	55.6
Ligand (PEP)	29.63 at 0.75 occupancy
**r.m.s.d from target values**	
Bond lengths (Å)	0.0100
Bond angles	1.3466
Dihedral angles	0.0749
Ramachandran	
Preferred (%)	97.55
Allowed (%)	1.84
Outliers (%)	0.61
**PDB Entry**	4UCG

## Results

### Interface Analysis of Interface and Choice of Mutation Site

The minor interface through which the tetramer is formed comprises primarily hydrophilic interactions, in contrast to the extensive hydrophobic interactions that contribute to the tight dimer interface (Fig 1). Of note is the single salt bridge between Arg126 and Glu27 from opposing tight dimers, which may play a role in determining the twist between tight dimers (Fig 1). *E*. *coli* DAH7PS shares a similar tetramer interface to that of *Nme*DAH7PS, and contains the same Glu-Arg salt-bridge. This salt bridge is not observed for *S*. *cerevisiae D*AH7PS, whose tetramer interface consists solely of hydrophobic interactions between four residues from each chain. This salt bridge was disrupted by substitution of Arg126 to a serine residue and this change was chosen to maintain the hydrophilicity of the protein surface.

The variant protein *Nme*DAH7PS^R126S^ was created using site directed mutagenesis and purified following the protocol developed for the wild-type protein (*Nme*DAH7PS^WT^). *Nme*DAH7PS^R126S^ had an expected mass of 77,300 Da, and showed similar thermal stability properties to the wild-type protein, with a melting temperature measured by differential scanning calorimetery of 47°C (*Nme*DAH7PS^WT^ displayed a melting temperature of 49°C) (S1 Fig).

### *Nme*DAH7PS^R126S^ Is Dimeric in Solution

**Fig 2:**
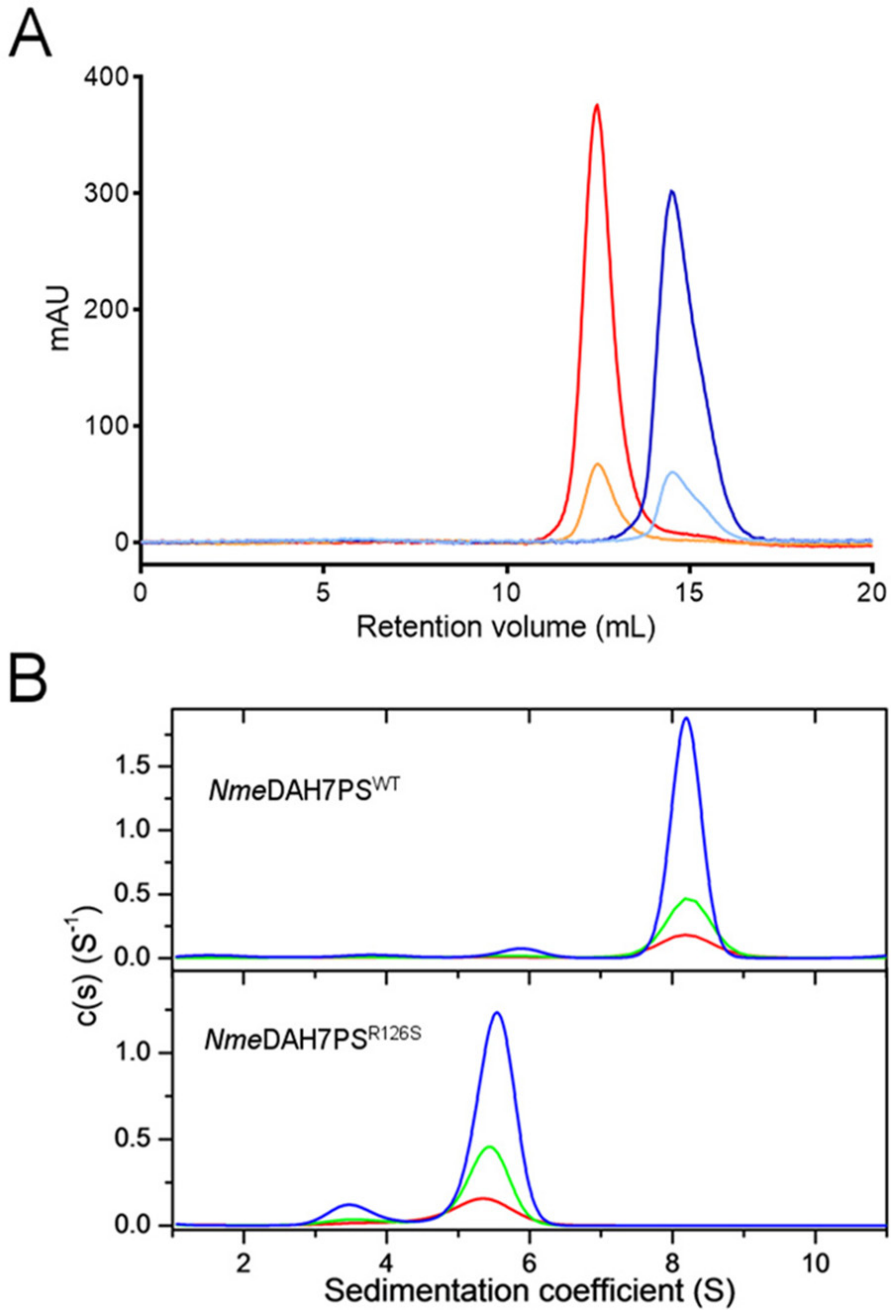
(A) Analytical SEC traces of *Nme*DAH7PS^WT^ and *Nme*DAH7PS^R126S^ at two different concentrations. *Nme*DAH7PS^WT^ 1 mg/mL (red) and 0.2 mg/mL (orange). *Nme*DAH7PS^R126S^ 1 mg/mL (blue) and 0.2 mg/mL (light blue). B. Calculated sedimentation coefficient c(s) distribution plot of wild-type *Nme*DAH7PS (top) and *Nme*DAH7PS^R126S^ (bottom). For both enzymes, the distribution of species for three different concentrations are shown: 0.2 mg/mL (red), 0.4 mg/mL (green) and 1.2 mg/mL (blue). The sedimentation velocity data, fit data and residuals from the fits are shown in S2 Fig.

Analysis of the quaternary structure assembly of the *Nme*DAH7PS^WT^ and *Nme*DAH7PS^R126S^ enzymes was carried out using both analytical size-exclusion chromatography (SEC) and analytical ultracentrifugation (AUC). SEC analysis revealed a clear difference in the solution behaviour of the two enzymes (Fig 2A). The wild-type protein gave a clear symmetrical peak shape corresponding to a mass of 150 kDa. Based on the theoretical molecular weight of a single wild type enzyme chain (38.7 kDa), this observation is consistent with a homotetrameric protein. Conversely, for *Nme*DAH7PS^R126S^ variant no tetrameric species was observed. An asymmetrical peak corresponding to a mass of ~66 kDa is consistent with the presence of a dimer under the conditions used for analysis. The same profile for each enzyme was observed when the samples were prepared for analysis and left at room temperature for 18 hours, indicating that the two enzymes observed form stable quaternary forms.

Sedimentation velocity experiments were carried out on *Nme*DAH7PS^WT^ and *Nme*DAH7PS^R126S^ enzymes. The absorbance versus radial position data for the *Nme*DAH7PS^WT^ and *Nme*DAH7PS^R126S^ variant are shown in Fig 2B. Fitting the data for *Nme*DAH7PS^WT^ to a continuous size distribution model indicates the presence of a major species with a standardised sedimentation coefficient of 8.1 S (Fig 2B). The calculated mass of this species is 141 kDa, which is consistent with the expected mass of a homotetrameric species (theoretical mass of 154.6 kDa). For *Nme*DAH7PS^R126S^, the fit indicated the presence of a major species with a standardised sedimentation coefficient of 5.7 S (Fig 2B). This predominant species has a calculated mass of 69 kDa, consistent with the expected molecular weight of the homodimeric assembly (77.3 kDa). At all three concentrations measured (0.4, 0.8, 1.2 mg/mL), a minor species with a sedimentation coefficient of 3.7 S, corresponding to an expected molecular weight of 36 kDa was observed, which is consistent with the molecular weight of monomeric *Nme*DAH7PS (38.7 kDa).

**Fig 3:**
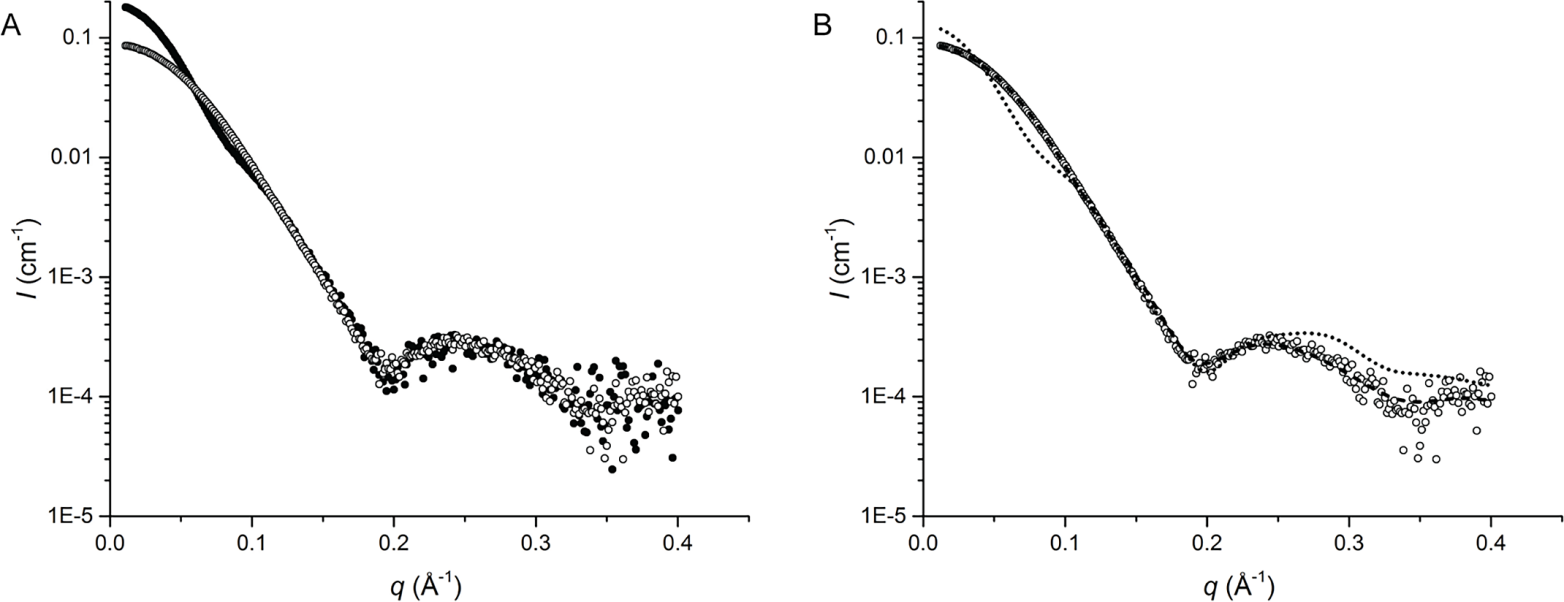
SAXS scattering profiles collected for *Nme*DAH7PS^WT^ and *Nme*DAH7PS^R126S^. (A) Overlay of the scattering data of *Nme*DAH7PS^WT^ (closed circles) and *Nme*DAH7PS^R126S^ variant (open circles) and (B) CRYSOL fits of the *Nme*DAH7PS^R126S^ variant data to the theoretical scattering data generated from the R126S tetramer (dotted line) and tight-dimer (dashed line). The discrepancies of the fit of the theoretical scattering of tetrameric and dimeric forms to the experimentally determined data (χ) were calculated as 22.80 and 0.67 respectively.

Small-angle X-ray scattering (SAXS) was employed to confirm the quaternary structure of *Nme*DAH7PS R126S in solution. The scattering profile collected for the *Nme*DAH7PS^R126S^ variant was compared to that collected for the wild-type enzyme (Fig 3, Table 2). The difference between the scattering intensities at low angles is indicative of a change in molecular weight and suggests these two enzymes adopt distinct oligomeric assemblies. The radius of gyration (*R*_g_) calculated from the scattering pattern of *Nme*DAH7PS^R126S^ variant using the indirect fourier transform function, (*P*(*r*)), was ~ 26 Å and the maximum diameter of the scattering particle, *D*_max_, was calculated as ~ 95 Å using GNOM [19]. These values are in close agreement with the tight-dimer generated from the *Nme*DAH7PS^R126S^ crystal structure (*R*_g_ of ~ 25 Å and *D*_max_ of ~ 86 Å). Similarly, CRYSOL [20] was used to directly compare the collected data to the hypothetical scattering from the *Nme*DAH7PS^R126S^ tetramer and dimer generated from the crystal structure. These data clearly show the *Nme*DAH7PS^R126S^ variant adopts a dimeric structure in solution comparable to that of the *Nme*DAH7PS^R126S^ tight-dimer (Fig 3B).

**Table 2:** SAXS parameters for *Nme*DAH7PS^WT^ and *Nme*DAH7PS^R126S^ variant.

Structural parameters	*Nme*DAH7PS^WT^	*Nme*DAH7PS^R126S^
*R*_g_ (Å)[from *P*(*r*)]	38 ± 1	27 ± 1
*R*_g_ (Å)[Guinier]	37 ± 1	27 ± 1
*D*_max_ (Å)[from *P*(*r*)]	110 ± 5	95 ± 5
*I*(0) (cm^-1^)[from Guinier]	0.113 ± 0.01	0.090 ± 0.01
Hydrated volume (Da^3^) [from *P(r)*]	224,000 ± 230	110,900 ± 110
Theoretical volume (Da^3^)[from CRYSOL]	222,900	101,300

### *Nme*DAH7PS^R126S^ Is Active

*Nme*DAH7PS^R126S^ was observed to be catalytically active with parameters that are comparable with those of the wild-type enzyme (Table 3). The apparent *K*_m_^PEP^ of *Nme*DAH7PS^R126S^ is 25 μM, which is similar to that observed for the wild-type enzyme (15 μM). The *Nme*DAH7PS^R126S^ displays a lower *K*_m_^E4P^ (13.4 μM) than the wild-type enzyme (37 μM). However, there is a noticeable decrease in specific activity for the dimeric variant. This implies that tetramerisation may have some influence on catalysis by *Nme*DAH7PS.

**Table 3:** Kinetic parameters of *Nme*DAH7PS WT and *Nme*DAH7PS^R126S^.

Organism	*K*_m(PEP)_ (μM)	*K*_m(E4P)_ (μM)	*k*_cat_ (s^-1^)	*k*_cat_/*K*_m(PEP)_	*k*_cat_/*K*_m(E4P)_
***Nme*DAH7PS**^**WT**^	15 ± 1	37 ± 2	27.1 ± 0.1	1.8	0.7
***Nme*DAH7PS**^**R126S**^	25 ± 1	13.4 ± 0.3	12.6 ± 0.1	0.5	0.9

Apparent *K*_m_ values were determined at high concentrations of the second substrate.

### *Nme*DAH7PS^R126S^ Shows Time-Dependent Loss of Activity

**Fig 4:**
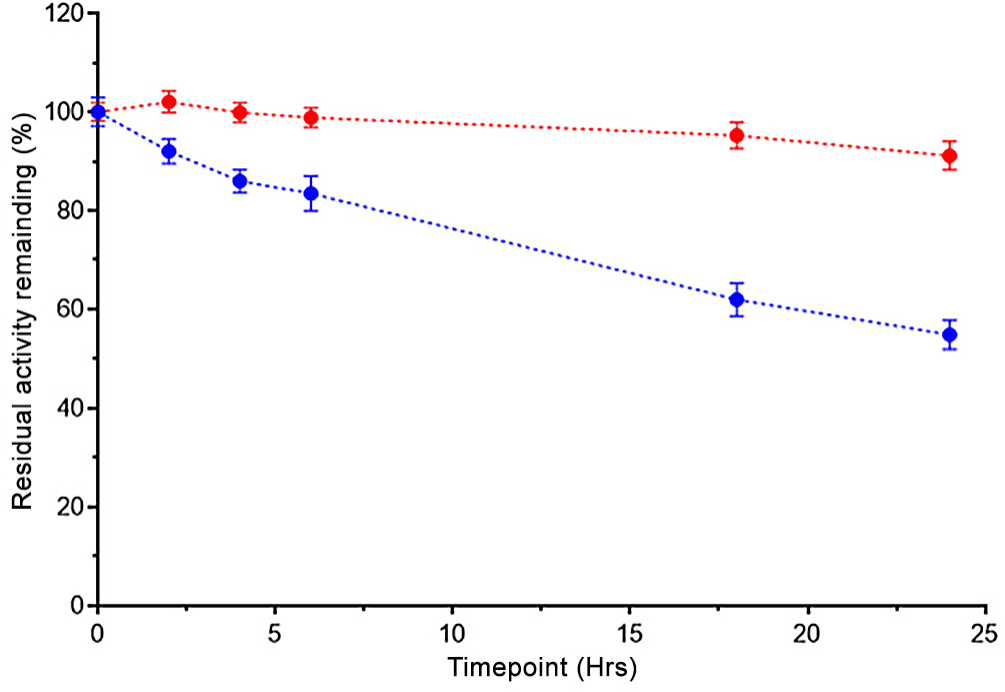
Catalytic activity of *Nme*DAH7PS^R126S^ (blue) *Nme*DAH7PS^WT^ (red) over 24 hour time period relative to maximum activity observed for each enzyme at the beginning of the experiment.

Comparison of stability of *Nme*DAH7PS^WT^ and the *Nme*DAH7PS^R126S^ was carried out by measuring the activity of the enzymes over a 24 hour period. *Nme*DAH7PS^WT^ lost ~10% catalytic activity, whereas the dimeric variant was far less stable and was observed to retain less than 60% of its activity after 24 hours (Fig 4).

### *Nme*DAH7PS^R126S^ Binds and Is Inhibited by Phe

**Fig 5:**
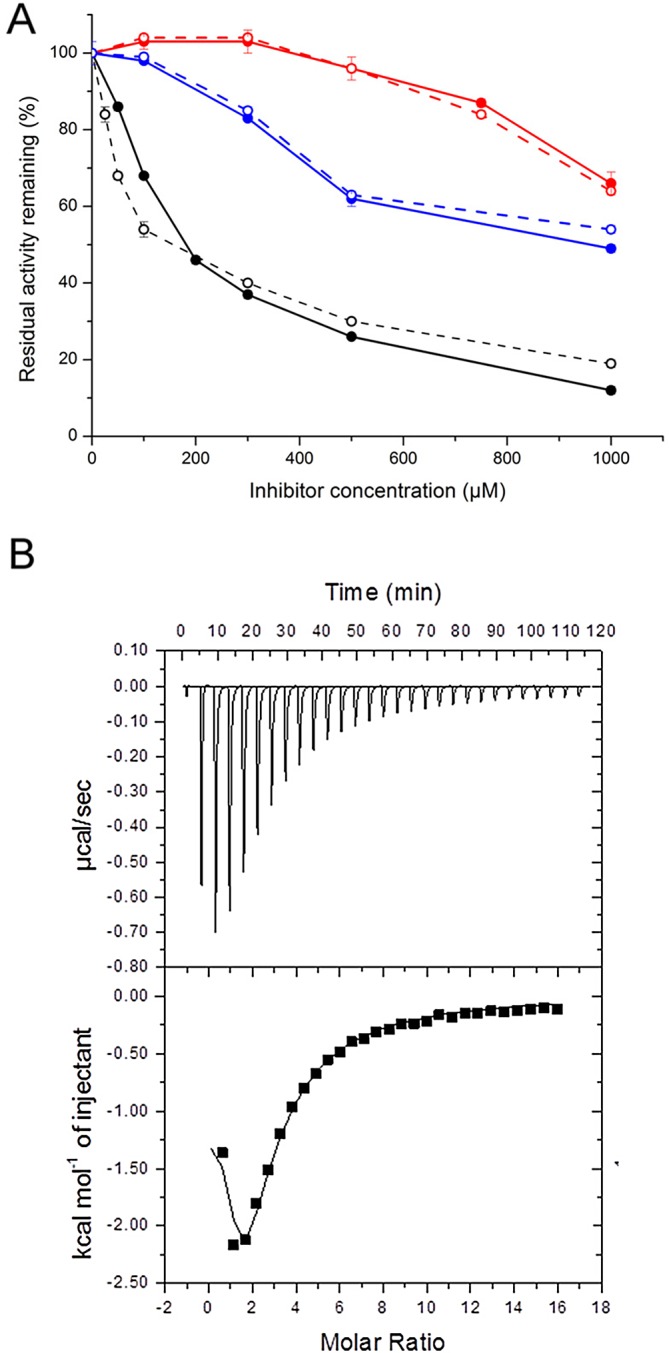
A. Effect of the aromatic amino acids, Phe (black), Tyr (red) and Trp (blue) on the catalytic activity of *Nme*DAH7PS^R126S^ (open circles) in comparison to data previously reported for the wild-type enzyme (closed circles) [12]. B. Isothermal titration calorimetry binding isotherm for *Nme*DAH7PS^R126S^ fit to a two-site sequential binding model.

As for the wild-type enzyme, *Nme*DAH7PS^R126S^ is inhibited to the largest extent by Phe and to a lesser extent by Tyr and Trp (Fig 5A). Isothermal titration calorimetry experiments were employed to illustrate that the affinity of the dimeric enzyme for Phe, was not altered upon mutation (Fig 5B). For *Nme*DAH7PS^R126S^ cooperativity was observed in the binding isotherm. The data were fitted to a two-site sequential model giving *K*_D_ values for *Nme*DAH7PS^R216S^ of 1.2 ± 0.7 μM for the first binding event and 33 ± 3 μM for the second binding event. This indicates similar Phe binding characteristics to those observed for *Nme*DAH7PS^WT^ [12].

### *Nme*DAH7PS^R126S^ Crystallises as a Tetramer

The crystal structure of the *Nme*DAH7PS^R126S^ variant was determined by X-ray diffraction. This structure was refined to a resolution of 2.0 Å and crystallised in an orthorhombic space group (*P*2_1_2_1_2_1_). Data collection and refinement statistics are shown in Table 1. This space group is different to *Nme*DAH7PS^WT^ (PDB code 4HSN), which crystallised in the monoclinic (*P*12_1_1) space group [12].

The asymmetric unit for *Nme*DAH7PS^R126S^ structure (PDB code: 4UCG) contains four chains interacting to provide a homotetramer, resembling the arrangement of wild-type *Nme*DAH7PS.

Like the wild-type enzyme, the *Nme*DAH7PS^R126S^ tetramer has two distinct interfaces between the chains. The tight dimer interface remains extensive, burying 14% of the surface area of the monomeric unit, but is smaller than *Nme*DAH7PS^WT^ (18%). The tetramer interface, on which the R126S substitution is located, is far smaller, burying 3.8% of the surface of the monomeric unit in *Nme*DAH7PS^WT^ and this is reduced to 3.4% for the *Nme*DAH7PS^R126S^. Superposition of the *Nme*DAH7PS^R126S^ homotetramer on the *Nme*DAH7PS^WT^ protein yields an RMSD of 0.61 Å (1328 Cα atoms), whereas superposition of *Nme*DAH7PS^R126S^ dimeric units on those of the wild-type protein gives RMSD values between 0.18–0.25 Å. These values are consistent with the small decrease in rotation of ~2°of the dimeric units with respect to each other at the tetramer interface, and this change may also account for the difference in space group between the *Nme*DAH7PS^R126S^ and *Nme*DAH7PS^WT^ structures.

According to PISA server calculations [16], the *Nme*DAH7PS^R126S^ crystal structure displays a theoretical solvation free energy gain (Δ*G*) for the formation of the tight dimer interface of -16.1 and -16.5 kcal/mol, correlating to the chain A-chain B and chain B-chain D interfaces respectively [16]. This value is less that what is observed for the *Nme*DAH7PS^WT^ enzyme, where the Δ*G* values are -20.1 kcal/mol and -18.1 kcal/mol. The lower values are indicative of a weakening of the tight dimer interface in *Nme*DAH7PS^R126S^.

**Fig 6:**
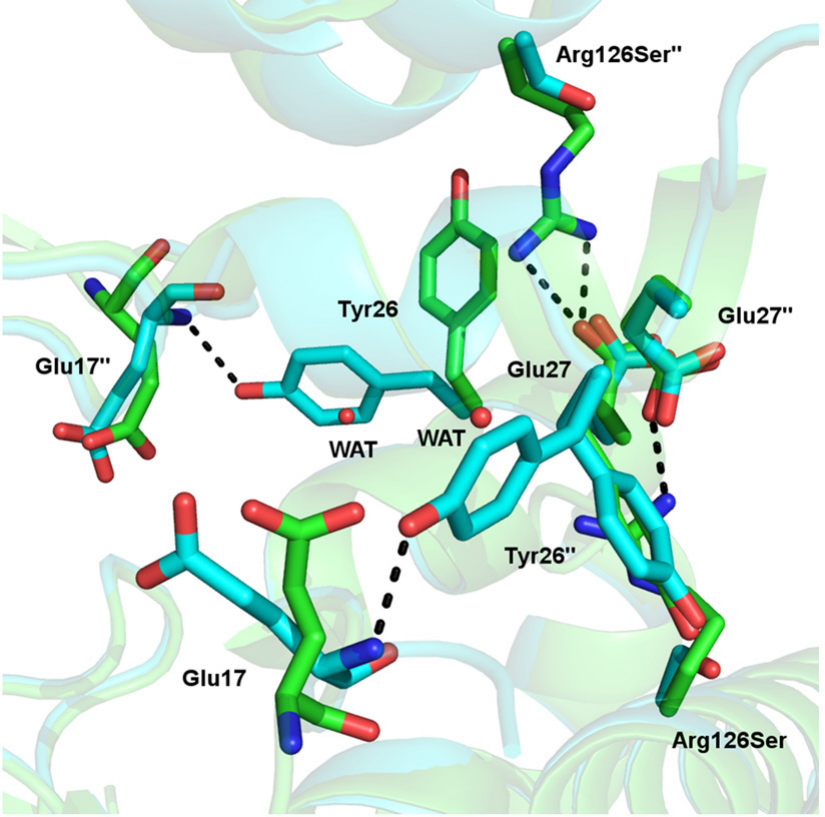
Tetramer interface of *Nme*DAH7PS^WT^ (green) (PDB code:4HSN) and *Nme*DAH7PS^R126S^ (cyan) indicating the variable positioning of Tyr26 in place of the conserved water molecule from the *Nme*DAH7PS^WT^. The loss of salt bridge between Glu27 and Arg126 for the *Nme*DAH7PS^R126S^ variant is also illustrated.

The Δ*G* for formation of the tetramer interface in *Nme*DAH7PS^R126S^ are -6.5 and -9.1 kcal/mol for the chain A-chain C and chain B-chain D interfaces respectively (interface interactions are shown in S3 Fig). Comparison of this with the Δ*G* for formation of the tetramer interface in *Nme*DAH7PS^WT^ of -6.9 and -7.0 kcal.mol, provides interesting insight into the structural asymmetry at the tetramer interface in *Nme*DAH7PS^R126S^. The structural asymmetry observed in the crystal structure of *Nme*DAH7PS^R126S^ may contribute to the in-solution dimerisation of this variant. This directly implicates the Arg126-Glu27 salt bridge in the formation of a symmetrical tetramer interface in *Nme*DAH7PS^WT^. Comparison of the surrounding residues in the crystal structure of *Nme*DAH7PS^R126S^ with that of *Nme*DAH7PS^WT^ illustrates this structural asymmetry. Tyr26, adjacent to Glu27, adopts a new, or alternate conformation in three of the four chains and in this new conformation, it forms a hydrogen bond with the backbone amine of Glu17. This position was previously occupied by a water molecule in the *Nme*DAH7PS^WT^ structure (Fig 6). Furthermore, His219, located at the centre of the tetramer, adopts several alternative conformations in the *Nme*DAH7PS^R126S^ structure as a result of this asymmetry.

## Discussion

A key feature of proteins is their tendency to form multimeric structures, usually homomers with even numbers of subunits all derived from the same gene, generating symmetry related interfaces, resulting in a functional biological assembly [31]. The DAH7PS family of proteins adopts a variety of homotetrameric forms, and like many other homotetramers, these tetramers are dihedral in nature and comprise dimers of dimers. Whereas the type Iα proteins share a common dimer subunit with the type Iβ group, they have a unique, small tetramer interface.

A single residue exchange in the small tetramer interface resulted in a solution- state dimer. The presence of this dimer and the absence of tetramer were confirmed by three independent solution-state techniques. The SAXS measurements also confirm that, as expected, the dimeric form adopts the assembly of the dimeric assembly of the parent protein. In contrast to these-solution state measurements, the protein crystallises as a tetramer. Some minor disruption and asymmetry has been introduced by the substitution indicating that this interface may be less stable. It should be noted that the dimeric form was observed by SAXS measurements even at relatively high concentrations, suggesting the crystal packing and crystallisation conditions favour the tetrameric form. Similar observations have been made with other related systems. The equivalent salt bridge of the *E*. *coli* Phe sensitive enzyme was disrupted by the mutation of Glu24 to Gln. This also resulted in a tetrameric crystalline form, however the enzyme was reported to be dimeric in solution, although no functional studies of this protein were provided [32]. Likewise, the tetrameric type Iβ enzyme from *Pyrococcus furiosus* has also been disrupted by mutation to form a mixture of dimeric and tetrameric forms in solution [11]. In this case the protein also crystallised as the tetramer.

The dimeric *Nme*DAH7PS retained its catalytic and allosteric properties. Catalysis was observed and both Phe binding and response was measured, revealing that the primary functional unit can be considered to be dimeric. The tetrameric form appears to provide advantage by a boost in catalytic rate and increased protein stability; the *Nme*DAH7PS^R126S^ enzyme lost significantly more catalytic activity over a long period than the wild-type tetrameric protein, and this instability may contribute to the reduction in *k*_cat_. Once again parallels can be drawn here from the studies with the *P*. *furiosus* DAH7PS. The thermal stability of this protein, derived from a hyperthermophilic source, was significantly reduced by disruption of the tetramer [11].

These studies may help shed light on the common evolution and evolutionary divergence of the DAH7PS proteins into the different classes. There is some evidence that dihedral homotetramers become established through the interaction of symmetrical dimers for which the largest interface is included. It has been shown that the symmetrical nature of the tetramer interface in *Nme*DAH7PS may be a contributing factor to tetramerisation. It is becoming increasingly clear is that both type Iα and type Iβ DAH7PS proteins are functional through a similar dimeric structure. Type Iα proteins differ in the extent of this interface, as the allosteric machinery including an N-terminal extension and a β-hairpin contributes to the interactions that form the extensive dimer interface and provide the binding site for Phe. Furthermore, it is notable that the binding of Phe to both the tetrameric and dimeric forms of *Nme*DAH7PS is inhibitory and follows a two-site sequential model suggesting that both the allosteric and catalytic function can be provided by the dimeric unit.

This investigation clearly illustrates the importance of a single salt-bridge at tetramer interface of *Nme*DAH7PS and has implications for the evolution of quaternary structure in this family of enzymes.

## Supporting Information

S1 Fig Differential scanning calorimetry showing thermal stability of *Nme*DAH7PS^WT^ and *Nme*DAH7PS^R126S^. (PDF)

S2 Fig Sedimentation velocity data for *Nme*DAH7PS^WT^ and *Nme*DAH7PS^R126S^. (PDF)

S3 Fig Interface analysis of *Nme*DAH7PS^WT^ and *Nme*DAH7PS^R126S^. (PDF)

## Abbreviations

BTP: 1,3-bis[tris(hydroxymethyl)methylamino]propane
DAHPS: 3-deoxy-d-*arabino-*heptulosonate 7-phosphate
DAH7PS: 3-deoxy-d-*arabino-*heptulosonate 7-phosphate synthase
E4P: erythrose 4-phosphate
PEP: phosphoenolpyruvate
Phe: phenylalanine
RMSD: root mean standard deviation
Trp: tryptophan
Tyr: tyrosine
