# Supplementary material for: The Functional Unit of Neisseria meningitidis 3-Deoxy-ᴅ-Arabino-Heptulosonate 7-Phosphate Synthase Is Dimeric
Source: PLoS One. 2016 Feb 1;11(2):e0145187. doi: 10.1371/journal.pone.0145187 (PMC4735112; doi:10.1371/journal.pone.0145187)
Supplement: S1 Fig — (PDF) [file pone.0145187.s001.pdf]

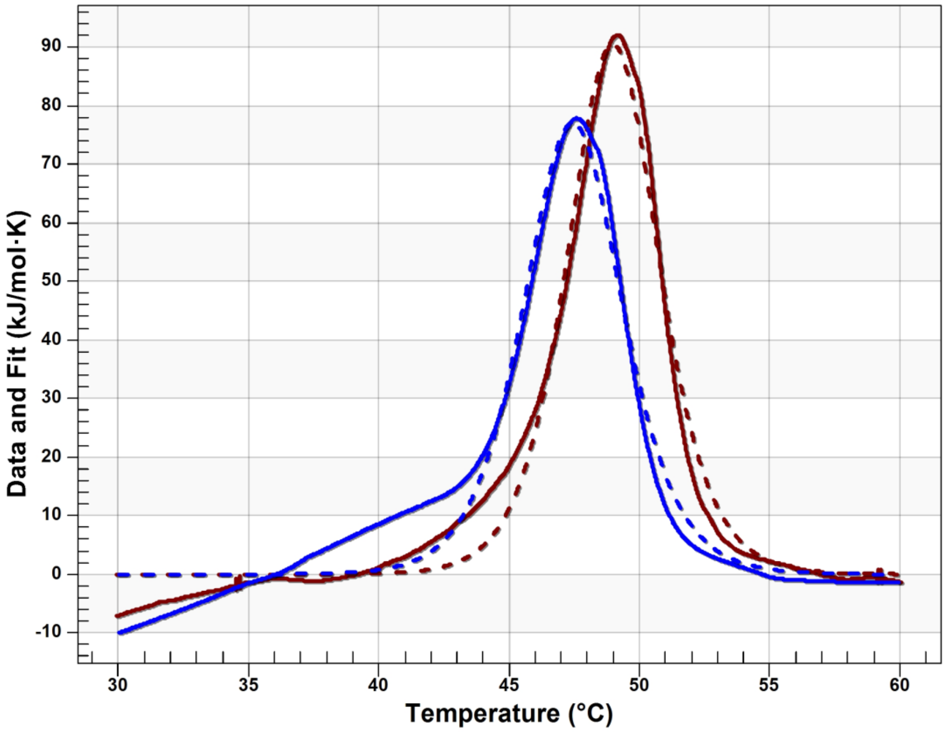

S1 Fig. Differential scanning calorimetry showing thermal stability of *Nme*DAH7PS^{WT} and *Nme*DAH7PS^{R126S}. *Nme*DAH7PS^{WT} (brown) and *Nme*DAH7PS^{R126S} (blue). Dashed lines indicate the fit of the model to the melting curve.
