# Supplementary material for: The Functional Unit of Neisseria meningitidis 3-Deoxy-ᴅ-Arabino-Heptulosonate 7-Phosphate Synthase Is Dimeric
Source: PLoS One. 2016 Feb 1;11(2):e0145187. doi: 10.1371/journal.pone.0145187 (PMC4735112; doi:10.1371/journal.pone.0145187)
Supplement: S2 Fig — (PDF) [file pone.0145187.s002.pdf]

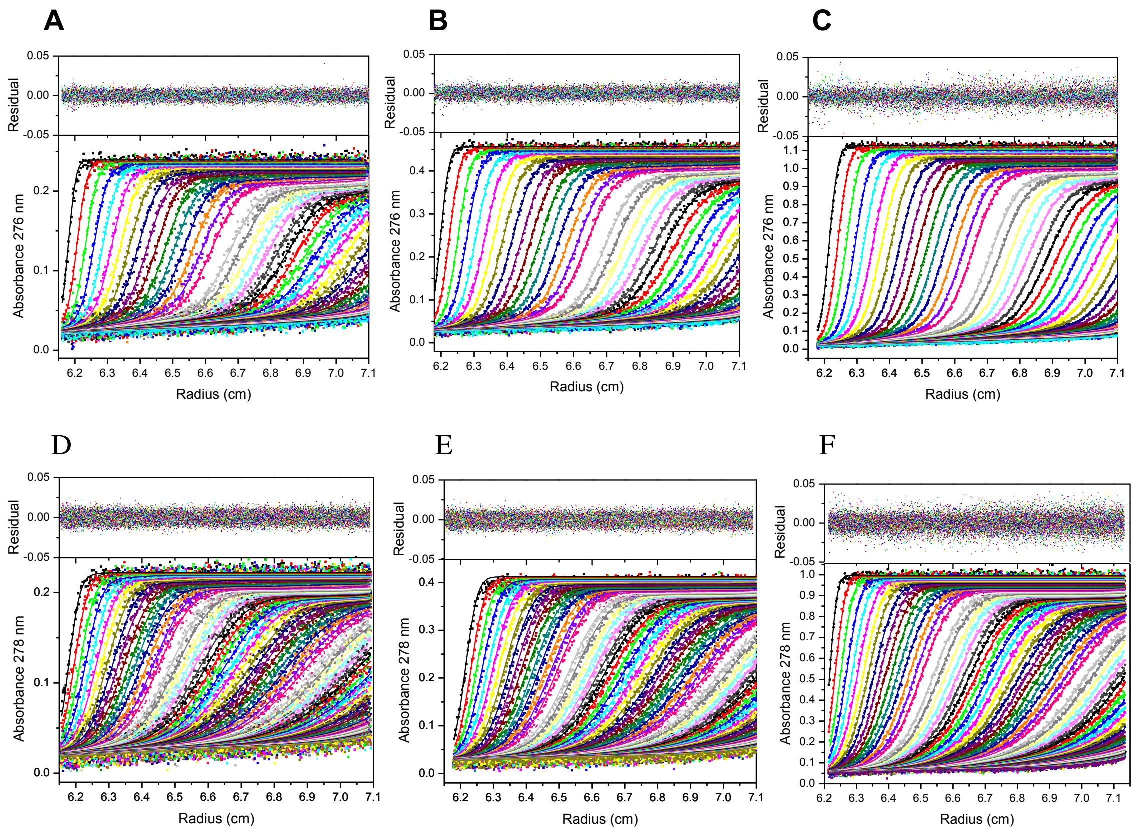

S2 Fig. Sedimentation velocity data for *Nme*DAH7PS^{WT} and *Nme*DAH7PS^{R126S}. Data collected at 20 ° C and 50,000 rpm. (A), (B) and (C) are the data from *Nme*DAH7PS^{WT} collected at 0.2, 0.4 and 1.2 mg.mL^{-1} and (D), (E) and (F) are the data from *Nme*DAH7PS^{R126S} collected at 0.2, 0.4 and 1.2 mg.mL^{-1}. The top panel in each section shows the sedimentation velocity data, the size-distribution best fit and residuals for the data fits.
