# Supplementary material for: The Functional Unit of Neisseria meningitidis 3-Deoxy-ᴅ-Arabino-Heptulosonate 7-Phosphate Synthase Is Dimeric
Source: PLoS One. 2016 Feb 1;11(2):e0145187. doi: 10.1371/journal.pone.0145187 (PMC4735112; doi:10.1371/journal.pone.0145187)
Supplement: S3 Fig — (PDF) [file pone.0145187.s003.pdf]

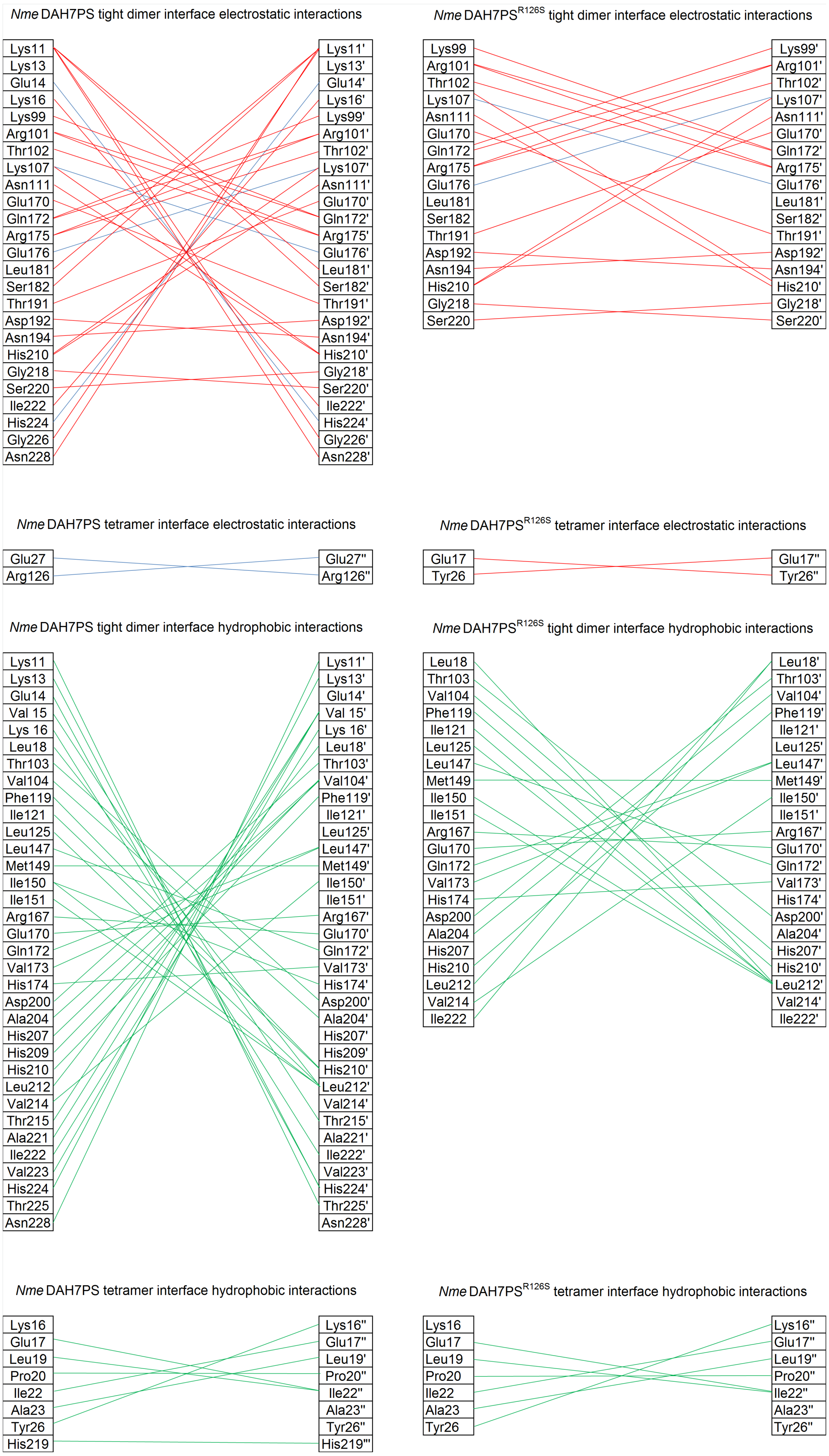

Nme DAH7PS tight dimer interface electrostatic interactions
Lys11 Lys13 Glu14 Lys16 Lys99 Arg101 Thr102 Lys107 Asn111 Glu170 Gln172 Arg175 Glu176 Leu181 Ser182 Thr191 Asp192 Asn194 His210 Gly218 Ser220 Ile222 His224 Gly226 Asn228
Lys11' Lys13' Glu14' Lys16' Lys99' Arg101' Thr102' Lys107' Asn111' Glu170' Gln172' Arg175' Glu176' Leu181' Ser182' Thr191' Asp192' Asn194' His210' Gly218' Ser220' Ile222' His224' Gly226' Asn228'
Nme DAH7PS^R126S tight dimer interface electrostatic interactions
Lys99 Arg101 Thr102 Lys107 Asn111 Glu170 Gln172 Arg175 Glu176 Leu181 Ser182 Thr191 Asp192 Asn194 His210 Gly218 Ser220
Lys99' Arg101' Thr102' Lys107' Asn111' Glu170' Gln172' Arg175' Glu176' Leu181' Ser182' Thr191' Asp192' Asn194' His210' Gly218' Ser220'
Nme DAH7PS tetramer interface electrostatic interactions
Glu27 Arg126
Glu27'' Arg126''
Nme DAH7PS^R126S tetramer interface electrostatic interactions
Glu17 Tyr26
Glu17'' Tyr26''
Nme DAH7PS tight dimer interface hydrophobic interactions
Lys11 Lys13 Glu14 Val 15 Lys 16 Leu18 Thr103 Val104 Phe119 Ile121 Leu125 Leu147 Met149 Ile150 Ile151 Arg167 Glu170 Gln172 Val173 His174 Asp200 Ala204 His207 His209 His210 Leu212 Val214 Thr215 Ala221 Ile222 Val223 His224 Thr225 Asn228
Lys11' Lys13' Glu14' Val 15' Lys 16' Leu18' Thr103' Val104' Phe119' Ile121' Leu125' Leu147' Met149' Ile150' Ile151' Arg167' Glu170' Gln172' Val173' His174' Asp200' Ala204' His207' His209' His210' Leu212' Val214' Thr215' Ala221' Ile222' Val223' His224' Thr225' Asn228'
Nme DAH7PS^R126S tight dimer interface hydrophobic interactions
Leu18 Thr103 Val104 Phe119 Ile121 Leu125 Leu147 Met149 Ile150 Ile151 Arg167 Glu170 Gln172 Val173 His174 Asp200 Ala204 His207 His210 Leu212 Val214 Ile222
Leu18' Thr103' Val104' Phe119' Ile121' Leu125' Leu147' Met149' Ile150' Ile151' Arg167' Glu170' Gln172' Val173' His174' Asp200' Ala204' His207' His210' Leu212' Val214' Ile222'
Nme DAH7PS tetramer interface hydrophobic interactions
Lys16 Glu17 Leu19 Pro20 Ile22 Ala23 Tyr26 His219
Lys16'' Glu17'' Leu19'' Pro20'' Ile22'' Ala23'' Tyr26'' His219'''
Nme DAH7PS^R126S tetramer interface hydrophobic interactions
Lys16 Glu17 Leu19 Pro20 Ile22 Ala23 Tyr26
Lys16'' Glu17'' Leu19'' Pro20'' Ile22'' Ala23'' Tyr26''

**S3 Fig.** Interface analysis of, left, *Nme*DAH7PS$^{WT}$ and right, *Nme*DAH7PS$^{R126S}$. Green lines indicate hydrophobic interactions at the interface, red lines indicate hydrogen bonds at the interface and blue lines indicate salt-bridges at the interface.
